# Structure and Temperature Dependence of Solder Layer and Electric Parameters in IGBT Modules

**DOI:** 10.3390/mi16091023

**Published:** 2025-09-05

**Authors:** Jibing Chen, Yanfeng Liu, Bowen Liu, Yiping Wu

**Affiliations:** 1 School of Mechanical Engineering, Wuhan Polytechnic University, Wuhan 420023, China; 2 School of Materials Science and Engineering, Huazhong University of Science & Technology, Wuhan 430074, China

**Keywords:** IGBT module, irregular temperature, thermal fatigue, microstructure, static parameters

## Abstract

IGBT high-power devices are subjected to various extreme working conditions for long periods and are affected by multiple loading conditions, inevitably leading to various aging and failure issues. Among them, the solder layer, as one of the weakest parts in the packaging structure of IGBT modules, has rarely been studied regarding its thermal fatigue characteristics and interface structure evolution behavior. In this work, a rapid temperature test chamber was used to conduct a thermal fatigue temperature cycling experiment on IGBT modules from −40 to 150 °C. The microscopic structural evolution behavior and the growth pattern of intermetallic compounds (IMC) during the solder layer’s thermal fatigue process of the IGBT modules were studied. At the same time, the changes in relevant static parameters of the IGBT after thermal cycling fatigue were tested using an oscilloscope and a power device analyzer, thereby clarifying the failure mechanism of the IGBT module. This provides a theoretical basis and data support for the thermal design and reliability assessment of IGBT modules.

## 1. Introduction

Electronic packaging includes technologies like high-lead solder, high-temperature alloy solder, low-temperature sintered nano Ag or Cu, and transient liquid phase diffusion bonding in terms of the types and forms of interconnect materials of chips [1,2]. Depending on the particular use of the packaging, these materials and procedures are employed in various ways, such as solder wire, solder sheet, and solder paste [3,4,5]. Extrusion, drawing, and forging are some of the processes used to create solder wire. Forging and extrusion are typically used to create solder sheets. Powder and organic solvent with a specific ratio are used to create solder paste [6,7]. To ensure that the joint has excellent thermal, electrical, and mechanical performance, it is also necessary to carefully consider factors such as the material’s strength, hardness, coefficient of thermal expansion, melting point, electrical resistance, thermal conductivity, and cost when choosing interconnection materials of chips [8,9,10].

The latest failure modes of IGBT modules are concentrated in three aspects: (1) bonding line failure: during power cycling, the aluminum wire undergoes mechanical fatigue due to thermal expansion differences, resulting in detachment or fracture, and high current density also accelerates aging through electrical migration; (2) solder layer fatigue: the difference in thermal expansion coefficients between the chip, solder, and substrate causes irregular thermal stress, leading to crack propagation in the solder layer and further exacerbating local temperature rise and delamination; (3) chip’s gate oxide breakdown and press-fit package substrate fracture: overvoltage at the gate causes breakdown of the oxide layer; however, press-fit modules alleviate solder layer fatigue, and mechanical stress is prone to causing substrate cracking [11,12,13]. This research focuses on studying the fatigue of the solder layer. Thus, choosing and utilizing suitable interconnection materials of chips is essential to guaranteeing the overall effectiveness and dependability of power device packaging. The electronics manufacturing industry in China is developing rapidly, and this is leading to ongoing research into solder materials for IGBT chips [14]. Gao et al. [15] conducted cavity simulation analysis on the solder layer of discretely packaged IGBTs by finite element analysis to study the various patterns of junction temperature and damage in the IGBT solder layer. Du et al. [16] proposed an improved structural thermal model based on the Cauer model that takes the impact of cavity rate into account. This model provides a more accurate description of the thermal conduction characteristics in the solder layer. Yang et al. [17] investigated the failure mechanism of the solder layer’s creep failure and fatigue interaction under the power cycle test (PCT). The results showed that the initial voids in the solder layer continuously collapsed, while new voids kept growing. That is, under short-time heating in the PCT, the solder layer would be deteriorated due to the generation and expansion of cracks. The reason for these results was that creep became the main factor causing the degradation of the solder layer. Therefore, under longer heating time and higher temperature, the failure of the solder layer is considered to be a complex creep failure and fatigue interaction failure process [18]. This research deals with temperature variations between high and low levels, rather than a constant PCT. Therefore, relatively speaking, fatigue failure constitutes the major factor.

The IGBT solder layer is essential to maintaining the dependability and functionality of the entire module because it acts as a bridge for thermal conduction and electrical connection. The two most frequent failure modes of the solder layer are heat cycling failure and power cycling failure, which are both primarily caused by a combination of different physical and chemical mechanisms [19,20]. These two failure modes both involve thermal stress caused by temperature changes during the switching on and off of the IGBT module, resulting from the mismatch in thermal expansion coefficients between the solder and different contact materials [21]. Under the action of such cyclic stress, microcracks will form and gradually grow within the solder layer or at the interface, leading to overall module failure when the damage reaches a certain extent, specifically manifested as fatigue failure.

Furthermore, during long-term operation, failure problems including chemical corrosion, void formation and growth, interfacial reactions, and creep may also have an impact on the solder layer’s dependability [22]. A physical failure mechanism known as creep occurs when solder material experiences continuous thermal stress and time-dependent deformation. This gradually reduces the solder joint’s mechanical strength and may eventually lead to mechanical failure. Cavities form and grow as a result of small bubbles caused by uneven thermal stress distribution within the solder layer, internal material defects, or manufacturing process defects. These cavities continue to grow and expand over time as the device is used, reducing the thermal conductivity and mechanical strength of the solder layer. Chemical corrosion is mainly due to the erosion of solder materials by chemicals, such as moisture and salts in the environment, resulting in the degradation of material properties [23]. For the interfacial reaction, the solder and the IGBT chip or substrate material interact chemically under high temperatures or particular operating conditions [24]. This result will cause the interfacial layer to embrittle or alter the material’s properties, which would compromise the solder layer’s overall stability.

In conclusion, this research conducted a thermal cycling temperature fatigue test on the solder layer of the IGBT module at temperatures ranging from −40 to 150 °C. The evolution law of the microstructure and IMC was analyzed, and the impact of thermal fatigue on the static parameters of the IGBT module was tested, to find the optimal working cycle temperature and resistance fatigue of the failure mechanism, which can provide technical support and theoretical guidance for the use of IGBT modules and related high-power devices.

## 2. Experiment and Methods

### 2.1. Design of Thermal Fatigue Experiment

**Figure 1:**
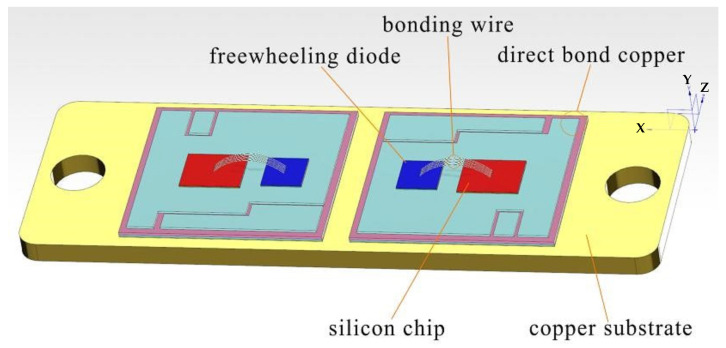
Schematic diagram of the internal structure of the IGBT module [25].

IGBT modules, as core components in the field of power electronics, are widely used in inverters, variable frequency drives, and electric vehicle drive systems, as shown in Figure 1. For these applications, IGBT modules frequently face drastic temperature changes due to their own heat generation and environmental temperature variations. These temperature fluctuations can affect the physical structure and electrical performance of the modules, thereby impacting their reliability and service life. Therefore, this study delves into the material stress changes and electrical performance parameter variations in the IGBT module packaging structure during repeated exposure to high and low temperatures through temperature cycling experiments. The principle is to set up a series of temperature cycling tests to simulate the extreme temperature conditions that the IGBT module may encounter in actual applications, ranging from −40 °C to +150 °C. Through repeated heating and cooling processes, the module is powered on at the end of the experiment to determine the changes in the electrical parameters of the IGBT module.

**Figure 2:**
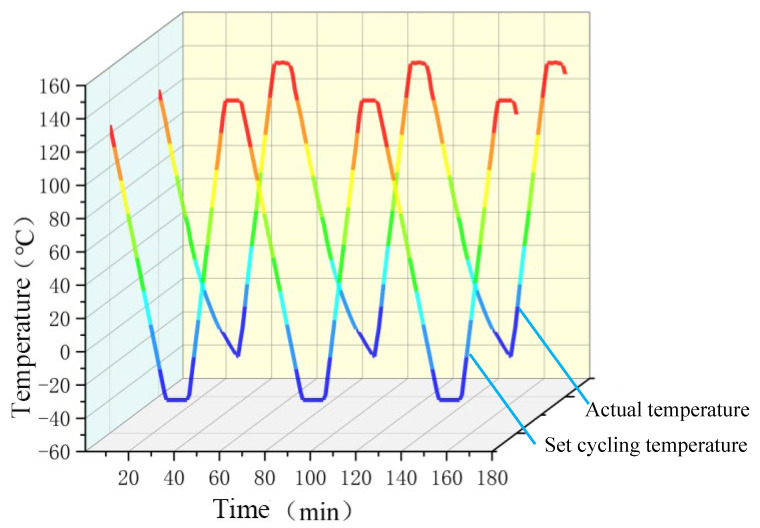
Temperature curves vs. cycling time of the solder layer in the IGBT module (Red to blue represents high temperature to low temperature).

Temperature cycling tests generally have three stages: the heating and cooling stage, the high-temperature holding stage, and the low-temperature holding stage. The design of this experiment refers to the standards [26]: GB/T 2423.22-2012 Environmental Testing Part 2. Test Methods: Temperature Cycling [27]. The experimental equipment used is the rapid temperature change test chamber (KC/WB-50L, Shanghai KaiCe Experimental Equipment Co., Ltd., Shanghai, China) [26]. According to the standards and the actual heating and cooling rates of the temperature change test chamber, the cycle period is one hour, the heating time is 15 min, the cooling time is 25 min, and the high- and low-temperature holding time periods are both 10 min, as shown in Figure 2. SCT is the set cycling temperature, and AT is the actual temperature inside the test chamber, with a total of 1000 cycles. During the experiment, the static parameters of the IGBT module were tested, and solder analysis was conducted at regular intervals.

In this research, an empirical study on the storage reliability of IGBT modules under extremely high- and low-temperature conditions was conducted. The IGBT modules are stored for an extended period at −40 °C and +150 °C, GB/T 2423.22-2012 [26] Environmental Testing for Electric and Electronic Products was the reference standard used with the rapid temperature change test chamber (KC/WB-50L). The experimental procedure is as follows: place the sample on the sample rack and put it into the test equipment for testing. Ensure that the samples are not stacked to fully expose them to environmental stress. After the test is completed, remove the samples and allow them to recover at normal room temperature for 2 h. Conduct electrical performance tests according to the electrical performance testing standards. The storage temperature is set according to the standard requirements: high temperature at 150~160 °C and low temperature from −60 to −50 °C, with a total.

### 2.2. Metallographic Preparation Techniques and Microscopic Observation

Metallographic preparation technology is an important technique, primarily used to observe and analyze the microstructure of metals and alloys. This technology involves a series of processes, such as grinding, polishing, and etching of the sample, achieving a sufficient level of surface smoothness to observe the internal microstructure of the material under a microscope. Metallographic preparation techniques are used to process the cross-section of IGBT modules that have been put through temperature cycling tests and high-low temperature storage tests. It will observe any changes in microstructural characteristics, the formation of defects in the solder layer, and any potential effects these processes may have on the IGBT modules’ performance.

**Figure 3:**
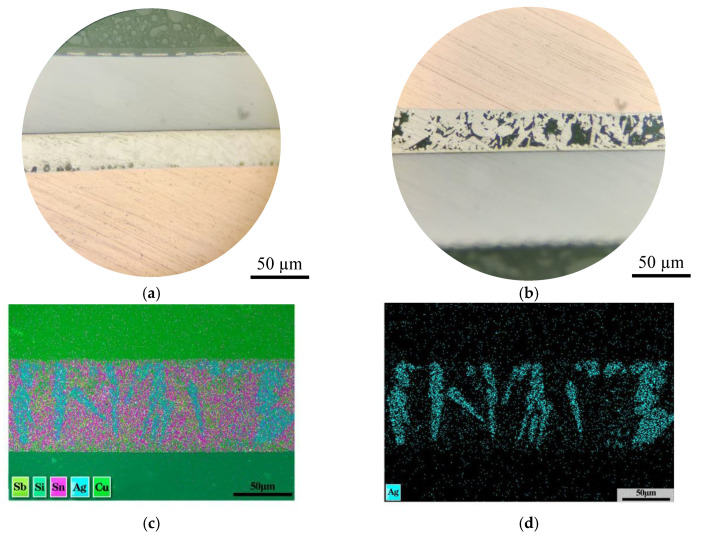
(**a**) Cross-sectional morphology of IGBT under microscope and (**b**) cross-sectional morphology after corrosion with 4% nitric acid alcohol solution; (**c**) IGBT cross-section EDS analysis image and (**d**) distribution of Ag in the solder layer.

Cross-section samples of the IGBT modules were longitudinally cut and treated with various fatigue experiments, including the solder layer, and were polished and finely ground using a small variable-speed electric grinder to ensure that the surface achieved a highly smooth mirror effect [27]. Figure 3a shows the morphology of the sample under a microscope. The cross-sectional morphology of the sample delineates the layers without any obvious bulging. To more clearly observe the effects of temperature cycling tests and high-low temperature storage experiments on the material, a 4% nitric acid-alcohol solution was used to etch the cross-section, as shown in Figure 3b. An energy dispersive spectroscopy (EDS) analysis was first performed on this cross-section because the exact type of solder used in this module is unknown. This is the EDS analysis spectrum in Figure 3c,d. The elemental composition and distribution characteristics of the solder used in this discrete IGBT module are determined by the spatial heterogeneity of the elements within the material, which is exhibited by the SnAgCu solder.

## 3. High- and Low-Temperature Storage and Temperature Cycling Experiments with Discrete IGBTs

### 3.1. Microstructure Analysis of Solder Layer Aging

**Figure 4:**
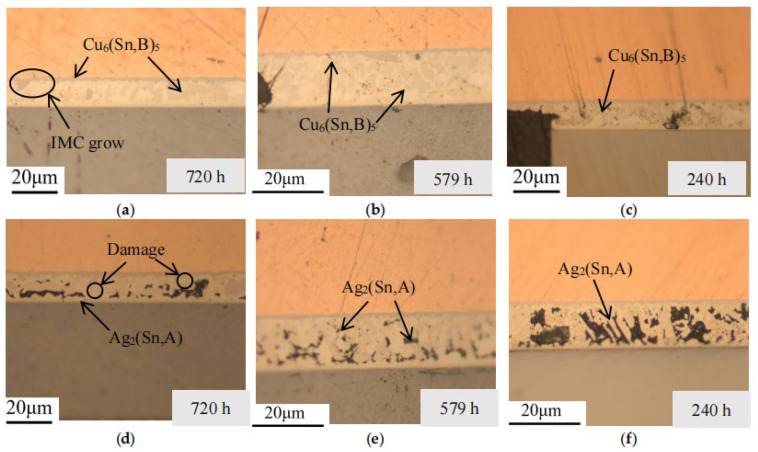
Cross-sectional metallographic images after high-temperature storage experiments. (**a**) Storage time: 720 h, (**b**) storage time: 579 h, (**c**) storage time: 240 h, (**d**) cross-section after corrosion for storage time of 720 h, (**e**) cross-section after corrosion for storage time of 579 h, and (**f**) cross-section after corrosion for storage time of 240 h.

**Figure 5:**
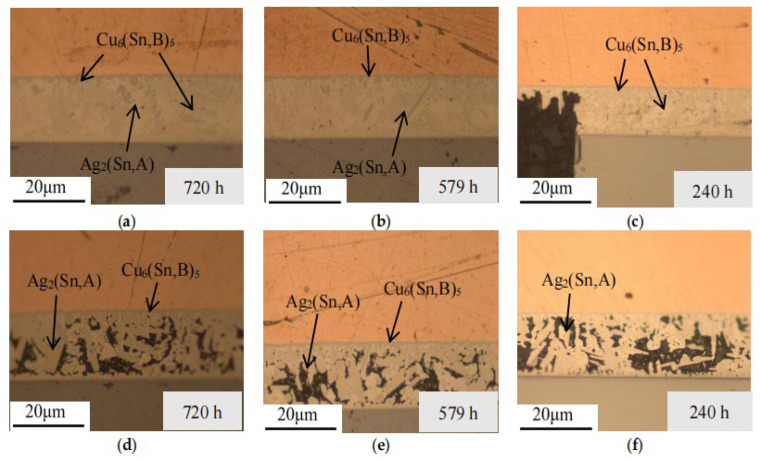
Cross-sectional metallographic images after low-temperature storage experiments. (**a**) Storage time: 720 h, (**b**) storage time: 579 h, (**c**) storage time: 240 h, (**d**) cross-section after corrosion for storage time of 720 h, (**e**) cross-section after corrosion for storage time of 579 h, and (**f**) cross-section after corrosion for storage time of 240 h.

**Figure 6:**
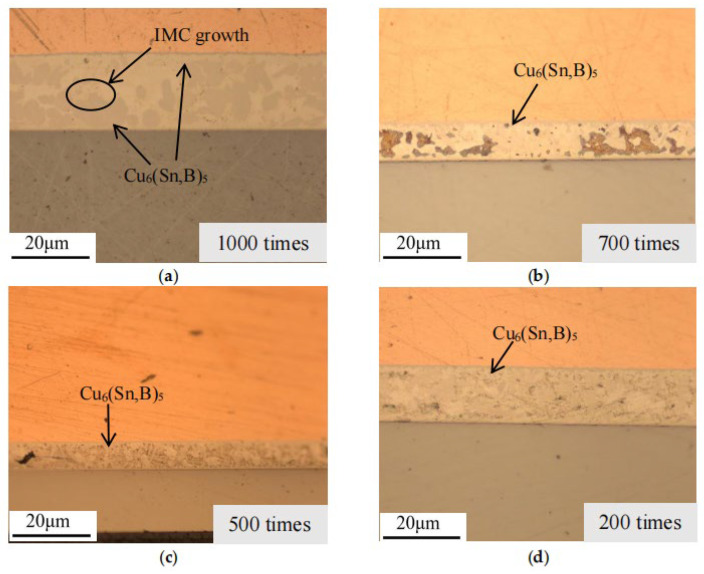
Cross-sectional metallographic images after temperature cycling experiments. (**a**) 1000 cycles, (**b**) 700 cycles, (**c**) 500 cycles, and (**d**) 200 cycles.

**Figure 7:**
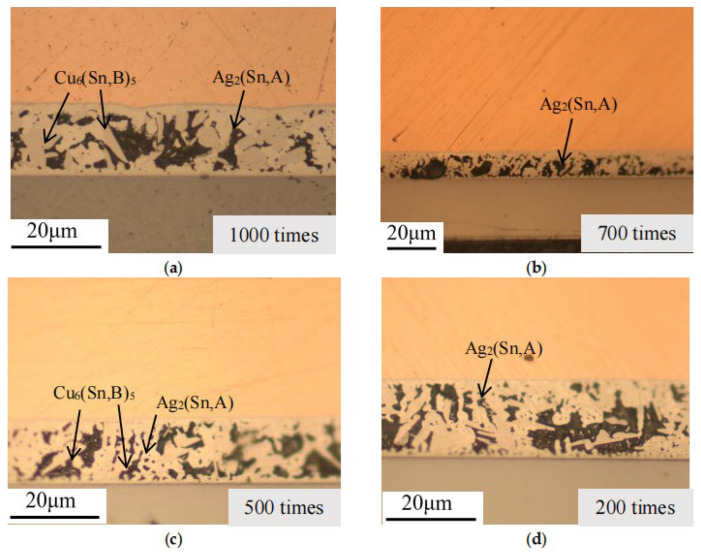
Cross-sectional corrosion microstructure after temperature cycling experiments. (**a**) 1000 cycles, (**b**) 700 cycles, (**c**) 500 cycles, and (**d**) 200 cycles.

To ascertain the phase composition, samples with varying experimental counts and time periods were prepared independently, imaged under an optical microscope, and combined with the solder layer morphology. The microstructure morphology evolution analysis results are illustrated in Figure 4, Figure 5, Figure 6 and Figure 7.

In the metallographic images, the three-layer structure of the IGBT, from top to bottom, consists of the copper layer, SAC (SnAgCu) solder layer, and Si chip layer. As observed in the images, after thermal fatigue testing, significant deformation occurs at the contact interface between the solder layer and the copper layer, showing an irregular, wavy pattern. According to previous studies [28,29], an intermetallic compound (IMC), Cu_6_Sn_5_, forms between the solder layer and the copper layer. With an increasing number of fatigue cycles, the IMC grows and thickens significantly, causing the interface to become uneven, and in some cases, delamination may occur (as shown in Figure 4a). This leads to a reduction in fracture toughness and increases the risk of material cracking. Within the solder, comparing Figure 3a and Figure 5a, and the referenced Figure 2, the temperature variations accelerate the atomic diffusion process, especially at elevated temperatures, promoting the reaction between copper, tin, and silver. This results in the aggregation and growth of the Cu_6_(Sn, B)_5_ phase. After the graphic dimension measurement, it was found that as the number of cycles increased from 200 times to 1000 times, the thickness of IMC increased from 5 µm to 18 µm. A thicker IMC layer may cause increased stress concentration during the expansion and contraction cycles, accelerating the formation and propagation of fatigue cracks.

The comparison of EDS spectra reveals that using a 4% nitric acid ethanol solution to etch the cross-section stains the Ag-containing phases. Ag_2_(Sn, A) appears as dots or stripes within the Sn matrix. After fatigue testing, the Ag_2_(Sn, A) particles inside the solder begin to coarsen with increasing aging time. Aggregation of Ag_2_(Sn, A) particles can be observed, while some are displaced or encapsulated (as shown in Figure 5e and Figure 7a). This occurs because Sn atoms react with Cu atoms diffused from the copper layer to form Cu_6_Sn_5_, which consumes part of the Sn atoms, causing them to be encapsulated by the faster-growing Cu_6_Sn_5_ phase.

Moreover, comparing these three rounds of experiments, the temperature cycling test, which simulates extreme temperature variation conditions, exerts greater stress and fatigue due to repeated thermal expansion and contraction. This effect is more severe than that caused by single-temperature experiments. Consequently, the temperature cycling test results in the most significant changes in the material’s microstructure, while the low-temperature storage test mainly affects the external packaging materials of the IGBT, such as increasing brittleness, and has relatively less impact on the solder layer.

### 3.2. The Impact of Fatigue Testing on the Static Parameters of IGBT

**Figure 8:**
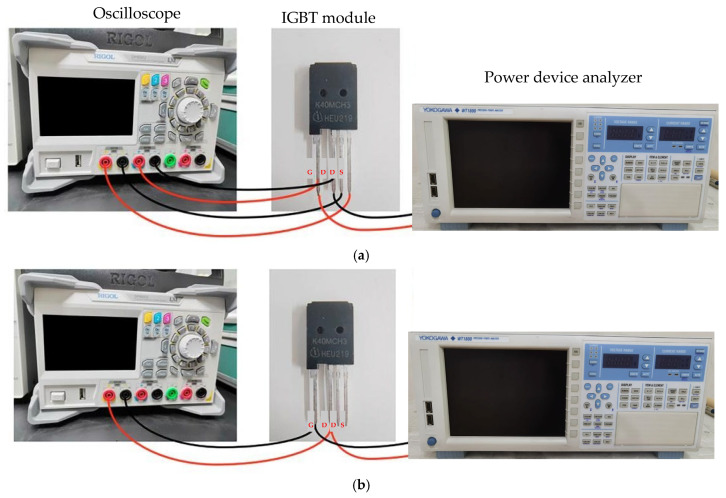
Extraction experiment: (**a**) Kelvin source-drain resistance (Rds-on), (**b**) forward voltage drop (Vds-on) of body diode.

Two electrical parameters of the experimental samples were tested after the temperature cycling and high/low temperature storage experiments on IGBTs: the forward voltage drop of the body diode (Vds-on) and the Kelvin source-to-power source resistance (Rds-on). The testing setup is shown in Figure 8a,b. The relationship between Rds-on, which measures the on-state resistance between the drain and source, and the device’s power loss efficiency is P = I^2^ × Rds-on, where *P* stands for power loss, *I* for current, and *R* for thermal resistance. The relationship between power loss efficiency and Rds-on mainly manifests in the on-state loss of the IGBT module. Rds-on is the equivalent resistance of the IGBT when it is conducting, and its value increases with the rise in junction temperature (JT), resulting in a significant increase in conduction loss (P = I^2^ × Rds-on), thereby reducing power efficiency. During the temperature cycling test, failure mechanisms such as solder aging and bond wire stress will further deteriorate the temperature drift characteristics of Rds-on, and exacerbate the static parameter drift. In summary, Rds-on is a key parameter for power loss efficiency, and its temperature drift characteristics are significantly affected by temperature cycling.

One of the main goals of high-performance device design is to minimize thermal losses under high-current operating conditions, which is indicated by a lower Rds-on value. Vds-on refers to the voltage drop across the IGBT’s body diode under forward bias, serving as a direct indicator of the internal voltage drop and switching efficiency in the conductive state. A decrease in Vds-on represents lower conduction losses and improved device performance. Monitoring the changes in these two parameters under extreme temperature conditions reveals the effects of temperature fluctuations on the IGBT’s structure and material properties, and can also predict the device’s performance and potential degradation in real-world applications. If significant changes in these parameters are detected after temperature stress testing, it indicates that the internal structure or material properties of the device have undergone subtle changes, which have important implications for the long-term stability and lifespan of the device.

In the process of the experiment, 11 discrete IGBT modules were selected, numbered, and grouped for testing. In the high- and low-temperature storage experiment, the modules were extracted at 7 time nodes based on storage duration for static parameter testing, with 3 time nodes selected for cutting the IGBTs to observe changes at the solder layer interface. In the temperature cycling experiment, modules were extracted at 6 cycle nodes for static parameter testing, and 4 nodes were selected for cutting to observe the solder layer interface changes. The experimental results are shown in Table 1 and Table 2.

Based on the analysis of the data from the charts, it can be concluded that during the high-temperature storage experiment, the elevated temperature accelerates the formation and diffusion of internal defects within the semiconductor material, such as lattice defects and impurity diffusion in the solder layer [30,31,32,33]. This increases the number of scattering centers, leading to a decrease in carrier mobility, which results in a gradual increase in Rds-on with longer storage durations. Defects in the solder layer lead instead to increased local temperatures, which in turn raise the device’s overall thermal resistance, thereby indirectly causing an increase in Rds-on due to enhanced carrier-carrier scattering.

Under low-temperature storage conditions, the increase in Rds-on is less pronounced. Notably, module 6 shows a decreasing trend in Vds-on, suggesting that low temperature may reduce carrier concentration and affect mobility [34,35,36,37]. Some of these effects may reverse when the material returns to normal temperature. However, if the low temperature causes permanent changes in the material or structure, such as damage to the solder layer, these effects may persist. The impact of temperature variation on IGBT performance is further confirmed by the temperature cycling experiment, more representative of real-world operating conditions. With temperature cycles increasing, the changes in Rds-on and Vds-on became more noticeable, with Vds-on displaying a maximum difference of 15 V. The most severe effects of temperature cycling were cumulative microstructural damage and variations in thermal stress, which resulted in material degradation and elevated internal resistance. These three sets of experiments’ observed variations in static parameters line up with the previously discussed microstructural alterations in the materials.

## 4. Conclusions

In this study, high and low temperature storage and temperature cycling experiments were conducted on discrete IGBT modules to analyze the effects of rapid temperature cycling conditions on the microstructure, IMC, and electrical parameters of the solder of the IGBT modules. The results show that as the cycle temperature, time, and number increase, the thickness of the microstructure layer increases, and the types of IMC and their layers increase, thereby increasing the brittleness between the layers. Under the drastic fluctuation of junction temperature (JT), the relevant electrical parameters will increase significantly, and the growth and coarsening of intermetallic compounds Ag_2_(Sn, A) and Cu_6_(Sn, B)_5_ in the solder layer will further exacerbate the risk of interface fracture and module failure, posing a serious threat to the long-term reliability of the IGBT module. Additionally, the long-term high-temperature storage would lead to significant increases in the Kelvin source-drain resistance (Rds-on) and the forward voltage drop of the body diode (Vds-on), with the maximum growth rate of Rds-on reaching approximately 12.5% and the maximum increase rate of Vds-on being about 400%. The impact of a low-temperature environment on the performance of the IGBT module and the encapsulation material is relatively small. The temperature cycling experiment is closer to the actual working conditions. This provides a theoretical basis and data support for the thermal design and reliability assessment of IGBT modules.

## Figures and Tables

**Table 1:** Kelvin source-drain resistance (Rds-on) test results (unit: Ω).

High-temperature storage experiment	Number	Initial Value	72 h	96 h	168 h	240 h	336 h	579 h	720 h
	2	1.52	1.52	1.54	1.55	1.55	1.62	1.64	1.71
	9	1.46	1.49	1.48	1.5	1.5	1.55	1.56	-
	10	1.5	1.52	1.54	1.56	1.56	-	-	-
Low-temperature storage experiment	Number	Initial value	72 h	96 h	168 h	240 h	336 h	579 h	720 h
	4	1.53	1.54	1.52	1.56	1.57	1.6	1.6	1.63
	6	1.5	1.5	1.54	1.56	1.55	1.58	1.58	-
	8	1.48	1.5	1.48	1.51	1.52	-	-	-
Temperature cycling experiment	Number	Initial value	50 times	100 times	200 times	500 times	700 times	1000 times	
	1	1.46	1.5	1.55	1.52	1.56	1.59	1.64	
	3	1.5	1.5	1.54	1.53	1.58	1.6	1.62	
	5	1.52	1.5	1.53	1.54	-	-	-	
	7	1.48	1.52	1.53	1.52	1.58	-	-	
	11	1.5	1.54	1.58	1.56	1.6	1.61	-	

“-” indicates that the sample was terminated prematurely due to time-dependent failure.

**Table 2:** Forward voltage drop of body diode (Vds-on) test results (unit: V).

High-temperature storage experiment	Number	Initial Value	72 h	96 h	168 h	240 h	336 h	579 h	720 h
	2	5.57	10.63	12.2	18.73	22.64	24.52	25.26	27.65
	9	3.31	11.24	15.16	19.86	20.85	23.03	25.86	-
	10	3.15	15.15	19.48	20.70	23.35	-	-	-
Low-temperature storage experiment	Number	Initial value	72 h	96 h	168 h	240 h	336 h	579 h	720 h
	4	5.81	3.98	3.63	10.08	10.08	9.28	11.37	12.03
	6	8.49	9.75	7.08	5.32	6.09	4.87	5.89	-
	8	4.23	4.77	5.06	6.39	7.95	-	-	-
Temperature cycling experiment	Number	Initial value	50 times	100 times	200 times	500 times	700 times	1000 times	
	1	15.42	18.95	21.52	23.54	25.04	26.78	29.37	
	3	14.78	19.43	20.16	23.16	26.57	28.85	30	
	5	9.83	12.49	17.88	19.82	-	-	-	
	7	5.44	9.57	13.55	16.89	21.43	-	-	
	11	12.05	15.22	17.63	21.93	24.97	27.49	-	

“-” indicates that the sample was terminated prematurely due to time-dependent failure.

## Data Availability

The original contributions presented in the study are included in the article, and further inquiries can be directed to the corresponding author.

## References

[1] Liu C. (2022). Advanced Pb-Free Interconnect Materials and Manufacture Processes to Enable High-Temperature Electronics Packaging. Ph.D. Thesis.

[2] Zhang H., Lee N.-C. (2020). High Temperature Lead-Free Bonding Materials—The Need, the Potential Candidates and the Challenges. Lead-Free Soldering Process Development and Reliability.

[3] Lutz J., Schlangenotto H., Scheuermann U., Doncker R.D. (2011). Semiconductor Power Devices.

[4] Amro R., Lutz J., Rudzki J., Thoben M., Lindemann A. Double-sided low-temperature joining technique for power cycling capability at high temperature. Proceedings of the 2005 European Conference on Power Electronics and Applications.

[5] Guth K., Siepe D., Görlich J., Torwesten H., Roth R., Hille F., Umbach F. New assembly and interconnects beyond sintering methods. Proceedings of the PCIM Europe International Exhibition & Conference for Power Electronics Intelligent Motion Power Quality.

[6] Scheuermann U., Wiedl P. Low temperature joining technology—A high reliability alternative to solder contacts. Proceedings of the Workshop on Metal Ceramic Composites for Functional Applications.

[7] Scheuermann U. Power module design for HV-IGBTs with extended reliability. Proceedings of the PCIM Europe 39th International Power Conversion Conference.

[8] Wu X., Yang X., Ye J., Liu G. (2025). Novel Prognostics for IGBTs Using Wire-Bond Contact Degradation Model Considering On-Chip Temperature Distribution. IEEE Trans. Power Electron..

[9] Wang J., Peng J., Cai S., Wang X. (2023). The effect of solvents on thermal stability of solder pastes in reflow process. J. Mater. Sci..

[10] Lee S., Yoo K.S. (2025). Design of Experiments for Optimizing Silver–Graphene Composite as a Conductive Paste. Korean J. Chem. Eng..

[11] Ismail N., Jalar A., Abu Bakar M., Ismail R., Ibrahim N.S. (2020). Effect of flux functional group for solder paste formulation towards soldering quality of SAC305/CNT/Cu. Solding Surf. Mt. Technol..

[12] Huang Z., Dong H., Chen B., Tang S., Ma Y., Liu W. (2023). Investigation of 1-hexanol base silver paste formula for low-temperature sintering. J. Mater. Sci. Mater. Electron..

[13] Zhao S., Tong Y., Wang C., Yao E. (2024). Challenges and progress in packaging materials for power modules with high operation temperature: Review. J. Mater. Sci.-Mater. Electron..

[14] Vianco P.T. (2022). A Review of Interface Microstructures in Electronic Packaging Applications: Brazing and Welding Technologies. JOM.

[15] Gao W., Guo Q., Peng Y., Ren M., Zhang B., Cai S. Influence of Solder Layer Void on Thermal Stability for Power Semiconductor Device. Proceedings of the 2019 20th International Conference on Electronic Packaging Technology (ICEPT).

[16] Du M., Guo Q., Wang H., Ouyang Z., Wei K. (2020). An Improved Cauer Model of IGBT Module: Inclusive Void Fraction in Solder Layer. IEEE Trans. Compon. Packag. Manuf. Technol..

[17] Zhao S., Yang X., Wu X., Liu G. (2025). Investigation on creep-fatigue interaction failure of die-attach solder layers in IGBTs under power cycling. IEEE Trans. Power Electron..

[18] Li L., Du X., Chen J., Wu Y. (2024). Thermal Fatigue Failure of Micro-Solder Joints in Electronic Packaging Devices: A Review. Materials.

[19] Hernes M., D’Arco S., Antonopoulos A., Peftitsis D. (2021). Failure analysis and lifetime assessment of IGBT power modules at low temperature stress cycles. IET Power Electron..

[20] Abuelnaga A., Narimani M., Bahman A.S. (2021). A Review on IGBT Module Failure Modes and Lifetime Testing. IEEE Access.

[21] Abueed M.A. (2020). Effects of Creep and Fatigue on the Reliability of SnAgCu Solder Joints in Thermal Cycling. Ph.D. Thesis.

[22] Alavi O., De Ceuninck W., Daenen M. (2024). Impact of Solder Voids on IGBT Thermal Behavior: A Multi-Methodological Approach. Electronics.

[23] Cui H., Zhou M., Yang C., Li J., Wang C. (2022). Influence mechanism of solder aging and thermal network model optimization of multi-chip IGBT modules. Microelectron. Reliab..

[24] Qin F., Zhang Y., An T., Zhou R. (2023). An Improved Thermal Network Model of Press-Pack IGBT Modules Considering Contact Surface Damage. IEEE Trans. Device Mater. Reliab..

[25] Chen J., Liu B., Hu M., Huang S., Yu S., Wu Y., Yang J. (2023). Study of the solder characteristics of IGBT modules based on thermal-mechanical coupling simulation. Materials.

[26] (2012). Environmental Testing—Part 2: Tests Methods—Test N: Change of Temperature.

[27] Ismail N., Atiqah A., Jalar A., Bakar M.A., Rahim R.A.A., Ismail A.G., Hamzah A.A., Keng L.K. (2022). A systematic literature review: The effects of surface roughness on the wettability and formation of intermetallic compound layers in lead-free solder joints. J. Manuf. Process..

[28] Tu C., Xu H., Xiao B., Lu J., Guo Q., Long L. (2022). Research on the Influence of Bond Wire Lift-Off Position on the Electro-Thermal Characteristics of IGBT. IEEE Trans. Electron Devices.

[29] Chen J., Deng E., Liu P., Yang S., Huang Y. (2021). The Influence and Application of Bond Wires Failure on Electrothermal Characteristics of IGBT Module. IEEE Trans. Compon. Packag. Manuf. Technol..

[30] Yang Y., Chen J., Liu B., Wu Y. (2024). Simulation and assessment of thermal-thermal coupling of welding materials in IGBT. Micromachines.

[31] Chen J., Chen J., Wang H., He L., Huang B., Dadbakhsh S., Bartolo P. (2025). Fabrication and development of mechanical metamaterials via additive manufacturing for biomedical applications: A review. Int. J. Extrem. Manuf..

[32] Shi Q., Chen J., Chen J., Liu Y., Wang H. (2024). Application of additively manufactured bone scaffold: A systematic review. Biofabrication.

[33] Hu M., Zhi S., Chen J., Li R., Liu B., He L., Yang H., Wang H. (2024). Restraint of intermetallic compound and improvement of mechanical performance of Ti/Al dissimilar alloy by rotary friction welding based on laser powder bed fusion. J. Manuf. Process..

[34] Takeuchi K., Higurashi E. (2025). Wafer Bonding of GaAs and SiC via Thin Au Film at Room Temperature. Micromachines.

[35] Zhang Y., Zhang J., Wang Y., Fang Y. (2022). Effect of Grain Structure and Ni/Au-UBM Layer on Electromigration-Induced Failure Mechanism in Sn-3.0Ag-0.5Cu Solder Joints. Micromachines.

[36] Chen J., Liu Y., Yang J., He L., Tang H., Li X. (2025). A review of development and applications of SiC power devices in packaging and interconnect technology. Solder. Surf. Mt. Technol..

[37] Du X., Chen J., She Y., Liu Y., Yang Y., Yang J., Dong S. (2023). Effect of process parameter optimization on morphology and mechanical properties of Ti6Al4V alloy produced by selective laser melting. Prog. Nat. Sci. Mater. Int..

